# Management of long-term cryptococcal meningitis neoformans in a surviving patient: A case report

**DOI:** 10.3389/fmed.2022.1035201

**Published:** 2022-12-07

**Authors:** Shijun Hu, Tao Liu, Shixiong Huang, Hui Liang

**Affiliations:** Department of Neurology, Hainan General Hospital, Hainan Affiliated Hospital of Hainan Medical University, Haikou, China

**Keywords:** cryptococcal meningitis, hydrocephalus, ventriculoperitoneal shunt, CSF pressure, standardized medical therapy

## Abstract

Cryptococcal meningitis is a common fungal infection of the central nervous system with high mortality and disability rates. A prominent clinical manifestation is persistent and severe cranial hypertension, which is one of the most critical outcome determinants in patients with cryptococcal meningoencephalitis. Herein, we report and discuss a case of cryptococcal meningitis treated by an inadequate course of medical therapy and placement of a ventriculoperitoneal shunt in a patient who survived for more than 10 years.

## Introduction

Cryptococcal meningitis is the most common fungal infection of the central nervous system (CNS) and has a high mortality rate; it is mainly caused by *Cryptococcus neoformans* or *Cryptococcus gattii* infection (1, 2). Cryptococcal meningitis can occur in AIDS patients and other immunocompromised persons. An estimated 223,100 cases of cryptococcal meningitis occur worldwide each year, resulting in approximately 181,100 deaths annually (3). Early detection and management of cryptococcal infection, a rapid reduction in fungal burden, and control of intracranial pressure can reduce mortality, whereas suboptimal antifungal treatment and immunological dysregulation can lead to treatment failure (4). Intracranial hypertension is a serious complication of cryptococcal meningitis, and cerebral edema caused by a rapid increase in intracranial pressure is an important predictor of a poor prognosis in the early stage of the disease (4, 5). More than half of patients with cryptococcal meningitis have an intracranial pressure exceeding 250 mmH_2_O, and aggressive control of intracranial pressure significantly improves survival odds (6, 7). The mechanism of intracranial hypertension may be mechanical obstruction of the outflow by Cryptococci that block the passage of CSF across the arachnoid villi; similarly, cryptococcal capsular polysaccharides can accumulate in the arachnoid villi and subarachnoid spaces, impairing the CSF drainage system (6). Herein, we report a case of cryptococcal meningitis that was treated with only fluconazole for several days and eventual ventriculoperitoneal shunt placement; the patient was infected with *Cryptococcus* for a long time without thorough treatment.

## Case description

A 34-year-old man who was previously healthy and who did not have a medical history or previous medical care was admitted to the hospital on 13 January 2011, due to intermittent fever for a month. The peak axillary temperature was approximately 38.5°C, and the patient reported progressively worsening headache, malaise, and decreased appetite and wasting, followed by bilateral lower limb malaise, walking instability, blurred vision and memory loss for 10 days prior to admission.

Initially, the patient was admitted to the Department of Infectious Diseases. On examination, the patient was thin, had lost 7.5 kg, exhibited diminished lower extremity muscle power in both legs (4/5), was positive for the Kernig sign, answered questions incoherently, had papilledema, and had poor memory. The results of other neurological and other system examinations were normal.

**FIGURE 1:**
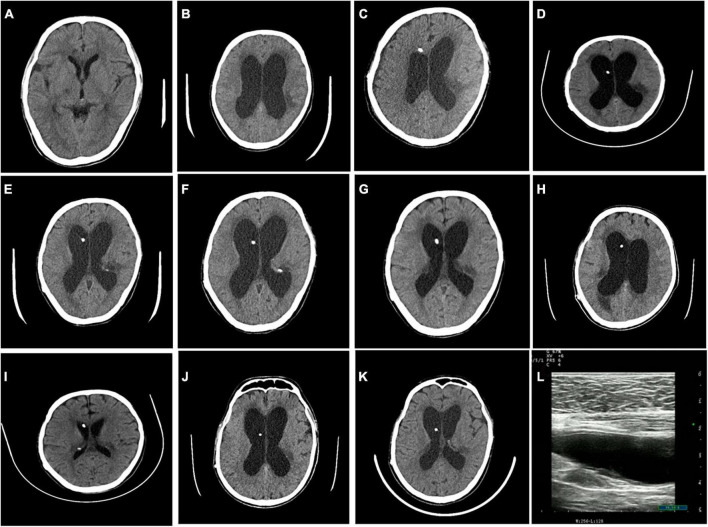
Cranial CT findings during the patient’s six hospitalizations. No hydrocephalus was observed during the first hospitalization **(A)**. Hydrocephalus was visible before the operation during the second hospitalization **(B)**, and hydrocephalus was relieved post-operatively **(C)**. Hydrocephalus was visible before the operation during the third hospitalization **(D)**, and hydrocephalus was relieved post-operatively **(E)**. Hydrocephalus was visible before the operation during the fourth hospitalization **(F)**, and hydrocephalus was relieved post-operatively **(G)**. Hydrocephalus was visible before the operation during the fifth hospitalization **(H)**, and hydrocephalus was relieved post-operatively **(I)**. Hydrocephalus was visible before the operation during the sixth hospitalization **(J)**, and hydrocephalus was relieved post-operatively **(K)**. Abdominal ultrasonography before surgery suggested an intra-abdominal encapsulated effusion **(L)**.

The patient was negative for HIV and exhibited normal liver function. A routine blood examination revealed a hemoglobin level of 86 g/L. The cerebrospinal fluid (CSF) was colorless and clear, the white blood cell (WBC) count was 69*10^6^/L, mononuclear cells were 85%, the glucose level was 0.82 mmol/L, the chloride level was 116.0 mmol/L, and India ink staining was positive (Table 1). Despite the CSF pressure exceeding 330 mmH_2_O, brain edema and hydrocephalus were not observed on cranial CT (Figure 1). CSF culture was negative.

**TABLE 1:** Cerebrospinal fluid results of three examinations.

Date	Pressure, mmH_2_O	Protein, g/L	Cell number, 10^6^/L	Mononuclear cells, %	Glucose, mmol/L	Chloride, mmol/L	mNGS, sequence number^a^	India ink staining
2011.01	>330	0.32	69	85	0.82	116.0	/	+
2019.02	>330	1.5	8	/	0.26	117.3	/	−
2020.12	52	4.45	138	84	0.95	114.5	12	−

^a^*Cryptococcus neoformans*-specific sequence.

The patient’s family refused to accept the administration of amphotericin B liposomes or amphotericin B because of concerns about the side effects and financial costs of the drug. The patient was treated with intravenous fluconazole (400 mg/d), and cranial pressure was treated with intravenous mannitol and glycerol fructose. After the above treatments were administered, the patient’s body temperature normalized, and his neck stiffness was relieved. The symptoms of cranial hypertension, such as decreased visual acuity, papilledema, and weakness in both lower limbs, were significantly relieved. The patient’s family refused to continue treatment because of financial difficulties, and the patient was discharged on 24 January 2011.

After discharge, the patient did not follow the doctor’s advice regarding regular follow-ups and oral drug treatment. The patient self-treated at home with only intermittent oral herbal medicines of unknown compositions, and his condition did not improve. The patient was unable to perform daily physical activities due to remaining cognitive dysfunction and weakness in the lower limbs.

8 years later, the patient showed an obvious decline in cognition, an unstable walking gait, incontinence, nausea, frequent vomiting, blurred vision, and no fever for 20 days. Head computed tomography (CT) suggested hydrocephalus (Figure 1), and the patient was subsequently admitted to the Department of Neurosurgery for ventriculoperitoneal shunt placement on 22 February 2019. The CSF was colorless and clear, with a protein concentration of 1.5 g/L, a WBC count of 8*10^6^/L, a glucose level of 0.26 mmol/l, and a chloride level of 117.30 mmol/L (Table 1). The CSF pressure exceeded 330 mmH_2_O before the operation (Table 1). CSF India ink staining and CSF culture were negative. The CSF results remained suggestive of intracranial infection. The patient’s symptoms improved significantly after surgery, so this patient refused further examination and treatment.

**FIGURE 2:**
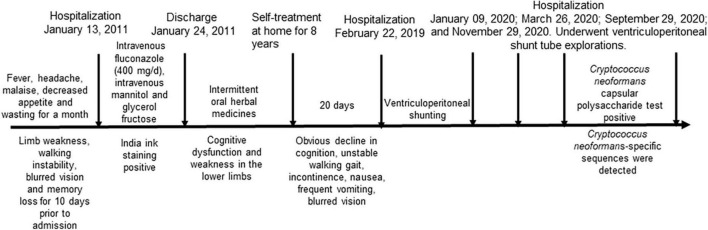
Timeline of the course of disease development.

Due to recurrent mechanical shunt obstruction, the patient underwent ventriculoperitoneal shunt tube explorations in the neurosurgery department on 09 January 2020; 26 March 2020; and 29 September 2020 (Figure 1). The patient underwent shunt tube exploration in the neurosurgery department again on 29 November 2020, due to hydrocephalus caused by mechanical shunt obstruction (Figure 1). Abdominal ultrasonography before the operation suggested an intra-abdominal encapsulated effusion with a size of approximately 80 mm * 20 mm * 31 mm; the boundary was clear, and there was no blood-flow signal. CSF examination revealed a protein concentration of 4.45 g/L, a WBC count of 138*10^6^/L, a glucose level of 0.95 mmol/L, and a chloride level of 114.50 mmol/L (Table 1). CSF India ink staining and CSF culture were negative, but the amount of *C. neoformans* capsular polysaccharide was 13.91 ng/ml in serum (lateral flow assay, normal range: 0–0.5 ng/ml). The results of metagenomic next-generation sequencing (mNGS) to detect pathogenic microbial DNA in the CSF revealed 12 *C. neoformans*-specific sequences; the procedure has been described elsewhere (8). The patient was then transferred to the neurology ward, and antifungal treatment was again recommended, but the patient’s family refused due to the excessive financial burden and possible serious side effects. Here, we present a detailed clinical timeline with the course of disease development (Figure 2).

## Discussion

The patient received a diagnosis of cryptococcal meningitis during the first hospitalization based on the symptoms of fever and cranial hypertension and positive CSF India ink staining, despite a negative CSF culture. The patient required several subsequent hospitalizations due to hydrocephalus and mechanical shunt obstruction, during which CSF protein concentrations, cell counts, glucose levels, and chloride level changes were suggestive of intracranial infection. *C. neoformans* capsular polysaccharide testing confirmed the presence of a large number of capsular polysaccharide antigens. Additionally, CSF mNGS detected *C. neoformans*-specific sequences, and no other secondary infections were identified. In China, *C. neoformans* is the most common pathogen of *Cryptococcus* infection (9), and this patient’s CSF mNGS results suggested that the pathogen was *C. neoformans*.

Current treatment guidelines recommend either amphotericin B plus flucytosine, amphotericin B liposomes plus flucytosine or fluconazole plus flucytosine during the induction phase for the treatment of cryptococcal meningoencephalitis in otherwise healthy people for at least 2 weeks (5, 10). Interestingly, the patient was treated with fluconazole for less than 2 weeks without consolidation and maintenance therapy but still survived for a long time. One reason for this is suggested by the fact that CSF culture results, which are an important indicator of the efficacy of induction-phase treatment, were consistently negative in this patient. Although fluconazole monotherapy can achieve good therapeutic effects, studies have shown that mortality in patients receiving this treatment regimen is more than 55% (11, 12), while the mortality rate in patients receiving the international standard induction treatment of amphotericin B plus flucytosine is 24.2% (13).

Although altered mental status, high CSF pressure, and positive India ink staining were considered risk factors for death due to cryptococcal meningitis in this patient, timely diagnosis and treatment may have contributed to this patient’s long-term survival (4, 14, 15).

In general, more than 50% of patients with cryptococcal meningitis have CSF pressures exceeding 250 mmH_2_O, and the risk of death can be reduced by controlling CSF pressure (7, 16). This patient was hospitalized several times for symptoms of cranial hypertension, including nausea, frequent vomiting, walking gait instability, incontinence, and blurred vision. The patient refused continuous antifungal therapy and had poor medical adherence, so a ventriculoperitoneal shunt was placed due to persistent cryptococcal infection. Studies have shown that ventriculoperitoneal shunt placement is a viable option in the presence of severe hydrocephalus with persistent cryptococcal infection (17, 18). The patient, despite benefiting from the alleviation of symptoms associated with persistent cranial hypertension by ventriculoperitoneal shunt placement, which may be one of the reasons for long-term survival, was hospitalized several times because of repeated mechanical shunt obstruction. According to a previous report, a high level of CSF polysaccharides may increase the risk of mechanical shunt obstruction (19).

It Is well known that capsular polysaccharides may still be present for an indeterminate period of time in patients who have recovered from *C. neoformans* infection, so a positive capsular polysaccharide test does not serve as strong evidence of active cryptococcal infection, especially when initial capsular polysaccharide levels are not known. However, the detection of high concentrations of capsular polysaccharides can often reflect the corresponding cryptococcal load (6). Little is known about how long the genetic material of pathogens can remain in the CSF of rehabilitated patients, so active cryptococcal infection could not be unequivocally diagnosed according to the CSF mNGS results in this patient, but it is extremely unlikely that cryptococcal genetic material from a previous infection could still be detected more than 10 years later. Additionally, a previous study suggested that when a *C. neoformans*-specific sequence read number was ≥2 on mNGS, a positive diagnosis of cryptococcal meningitis could be made, as the sensitivity and specificity were 76.92 and 99.52% (20), respectively. CSF glucose and chloride level changes in this patient also strongly suggested the presence of this specific intracranial infection.

Previous studies have reported that more than 65% of patients with cryptococcal meningitis in China are immunocompetent (21). However, recent findings have revealed that some underlying immunogenetic functional defects may exist in these immunocompetent individuals (22, 23). This patient could have been characterized in the past as immunocompetent but did not have the particular test results needed to clarify the presence of some level of immunodeficiency.

To the best of our knowledge, the longest duration of infection following shunt placement was more than two decades (24), but persistent, long-term cryptococcal meningitis has rarely been reported. The length of time that the patient received antifungal therapy and the remaining symptoms after discharge implied that this patient’s cryptococcal meningitis was probably never completely cured. It did not resemble recurrent cryptococcal infection; rather, it is likely that this patient experienced persistent cryptococcal infection. To our knowledge, this is the first case of persistent infection with long-term survival. The reason for this may be due to a somewhat delicate immune response balance between the fungus and the host (4). This immune response may not be sufficient to completely clear the fungus, but it may be sufficient to prevent the fungus from causing extensive injury to the host.

In conclusion, in patients with cryptococcal infection who have limiting conditions that preclude the use of internationally standard treatment options, active intracranial pressure management, such as dehydrating drugs, lumbar puncture, or ventriculoperitoneal shunt placement, may effectively improve clinical symptoms and prolong survival time.

## Data availability statement

The original contributions presented in this study are included in the article/supplementary material, further inquiries can be directed to the corresponding author.

## Ethics statement

Ethical review and approval was not required for the report of a single clinical case here in accordance with the local legislation and institutional requirements. The patients/participants provided their written informed consent to participate in this study.

## Author contributions

All authors listed have made a substantial, direct, and intellectual contribution to the work, and approved it for publication.
